# Visual process maps to support implementation efforts: a case example

**DOI:** 10.1186/s43058-020-00094-6

**Published:** 2020-11-25

**Authors:** Jennifer Kononowech, Zach Landis-Lewis, Joan Carpenter, Mary Ersek, Robert Hogikyan, Cari Levy, Ciaran Phibbs, Winifred Scott, Anne E. Sales

**Affiliations:** 1 Center for Clinical Management Research, VA Ann Arbor Healthcare System, 2800 Plymouth Rd, Ann Arbor, MI 48109 USA; 2 University of Michigan Medical School, 300 N. Ingalls Street, Ann Arbor, MI 48109 USA; 3 Corporal Michael J. Crescenz VAMC, 3900 Woodland Avenue, Philadelphia, PA 19104 USA; 4 School of Nursing, University of Pennsylvania, 418 Curie Blvd, Philadelphia, PA 19104 USA; 5 VA Ann Arbor Healthcare System, 2215 Fuller Road, Ann Arbor, MI 48105 USA; 6 Eastern Colorado Health Care System, 1055 Clermont St, Denver, CO 80220 USA; 7 School of Medicine, University of Colorado Anschutz Campus, 13001 E. 17th Pl, Aurora, CO 80045 USA; 8 Geriatrics and Extended Care Data and Analysis Center, VA Palo Alto Health Care System, 3801 Miranda Ave, Palo Alto, CA 94304 USA; 9 Department of Pediatrics, Stanford University School of Medicine, 725 Welch Road, Palo Alto, CA 94304 USA

**Keywords:** Long-term care, Goals of Care conversations, Process maps

## Abstract

**Background:**

Process mapping is often used in quality improvement work to examine current processes and workflow and to identify areas to intervene to improve quality. Our objective in this paper is to describe process maps as a visual means of understanding modifiable behaviors and activities, in this case example to ensure that goals of care conversations are part of admitting a veteran in long-term care settings.

**Methods:**

We completed site visits to 6 VA nursing homes and reviewed their current admission processes. We conducted interviews to document behaviors and activities that occur when a veteran is referred to a long-term care setting, during admission, and during mandatory VA reassessments. We created visualizations of the data using process mapping approaches. Process maps for each site were created to document the admission activities for each VA nursing home and were reviewed by the research team to identify consistencies across sites and to identify potential opportunities for implementing goals of care conversations.

**Results:**

We identified five consistent behaviors that take place when a veteran is referred and admitted in long-term care. These behaviors are assessing, discussing, decision-making, documenting, and re-assessing.

**Conclusions:**

Based on the process maps, it seems feasible that the LST note and order template could be completed along with other routine assessment processes. However, this will require more robust multi-disciplinary collaboration among both prescribing and non-prescribing health care providers. Completing the LST template during the current admission process would increase the likelihood that the template is completed in a timely manner, potentially alleviate the perceived time burden, and help with the provision of veteran-centered care.

**Supplementary Information:**

The online version contains supplementary material available at 10.1186/s43058-020-00094-6.

Contributions to the literature

- This is a pragmatic example of process mapping visualization approaches, which is lacking in the literature that is more focused on the theoretical use of process maps.
- These findings contribute to the recognized gaps in the literature, including ascertaining the usefulness of process mapping and demonstrating how it supports implementation of a national initiative.
- We also discuss steps beyond process mapping, to understand how workflow fits into assessment of barriers and facilitators to implementation.

## Background

Process mapping or visualization is an important approach to understanding how behaviors and activities need to change to support implementation of new practices [1–3]. We provide a brief case study illustrating approaches to making visual process maps to support implementation efforts. Our case example focuses on the implementation of goals of care conversations in long-term care settings in the Veterans Health Administration in the USA. More detail on the case example is given in Additional file 1, including the background of the initiative as well as the context of the implementation work. We also described the protocols and plans for our work in a protocol paper [4].

Briefly, the purpose of goals of care conversations (GoCC) is to elicit the patients’ values and treatment preferences either from the patient directly or, in the case of patients who lack capacity, from their designated surrogate decision-maker. The Veterans Health Administration (VHA), the largest integrated healthcare delivery system in the USA, prioritized conducting GoCC and documenting treatment preferences of seriously ill veterans. The aim of updated guidance for this work, called the Life-Sustaining Treatment Decisions Initiative (LSTDI), is to promote personalized, proactive, patient-driven care for veterans with serious illness.

The initiative has an associated LST progress note and order set within the VHA’s electronic medical record system. The progress note must be completed by licensed prescribing providers (providers who are credentialed to write orders, including prescriptions and other orders for care) and documents a GoCC with seriously ill veterans. The template is used to document decisions about initiating, limiting, or discontinuing life-sustaining treatments. Many clinicians, not just licensed prescribing providers, have a role in conducting and documenting GoCCs. Registered nurses, social workers, psychologists, and chaplains may have the initial conversation with a veteran to better understand his/her values and goals. Medical doctors (MDs), doctors of osteopathy (DOs), physician assistants (PAs), and nurse practitioners (NPs) explain a veteran’s diagnosis, prognosis, and establish an LST plan with the veteran and/or their surrogate; as licensed prescribing providers, this group of clinicians are responsible for completing and signing the LST progress note and order set.

Community living centers are units within VA that were previously known as nursing home care units [5]. The work we describe here was all done in VA CLCs. Before veterans are admitted into a CLC unit, they must be assessed to determine if the services provided by the CLC are appropriate. Different approaches to admission decision-making are used across VHA, depending on the care needs of the veteran, bed availability, and local policy. Once admitted, residents are evaluated using the minimum data set (MDS 3.0), a mandatory national tool that was designed to standardize assessment and determine care needs in VA and non VHA nursing homes [6].

Our program is called the Long-Term Care Quality Enhancement Research Initiative (referred to as the LTC QUERI) and is a quality improvement (QI) project in collaboration with VHA operational partners to examine approaches that enhance implementation of the LST progress note and order set in CLCs across the VA. It has been deemed non-research quality improvement by the VA Ann Arbor Healthcare System Research and Development Committee. The goal of this paper is to describe typical admission processes at different sites to create visual maps of these processes and identify behaviors and activities that can be modified to support implementation and use of GoCCs.

## Methods

Process maps show the steps and flow of a process and are used in quality improvement to examine current processes and identify areas to intervene to improve quality [7]. They allow clinicians and administrators to observe and understand patients’ experiences in the healthcare system and to understand specific behaviors that constitute workflow [8]. Our goal was to determine potential points for intervention and implementation in the admission process for completion of a GoCC and LST template and order set. We used a sequential flow diagram, presenting the steps in a process sequentially in the order in which tasks are accomplished [9].

Two to four LTC QUERI team members completed site visits to six CLCs in one Midwestern Veterans Integrated Service Network (VISN) between October 2015 and May 2016. These six sites were chosen because of their proximity to the LTC QUERI staff based in Ann Arbor, MI. The initial goal of the site visit was to develop rapport with a site champion who were identified as CLC leaders who agreed to be liaisons for the project. In addition to the site champion, we also met with and interviewed facility leadership and team members from nursing, quality management, restorative care, and social work. Site visits typically lasted 1 day each and included a combination of one-on-one meetings and group discussions. We conducted semi-structured interviews with the site champion and other CLC staff members to understand how veterans are currently admitted into the CLC and to identify existing or potential process changes that would facilitate conducting GoCC and documenting veterans’ LST preferences.

One team member (AES) created two initial process maps following our early site visits after analyzing the site visit notes. This map was circulated to the research team for feedback and refinement. For later site visits, the project manager (JK) took the lead in drafting the process maps for the remaining four sites after receiving training from AES in process map methods. These process maps were created by completing a careful review of the initial process maps alongside the later site visit notes. Similarities and differences among sites were identified and reflected in the process maps. After maps were drafted, they were analyzed for opportunities for process improvement related to implementing GoCCs and LST template completion. We used other information from the interviews to note potential barriers and facilitators to implementation, using the Tailored Implementation for Chronic Diseases checklist [10].

## Results

**Fig. 1:**
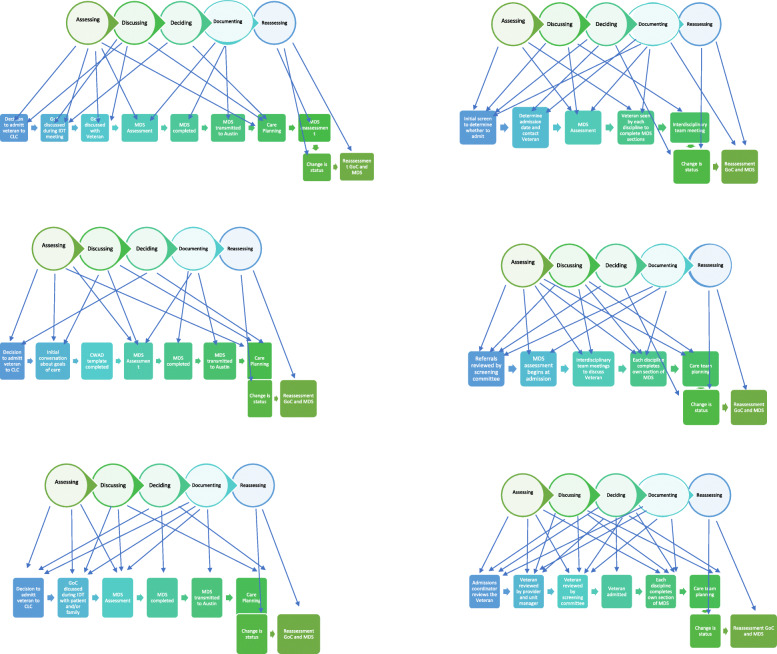
Process maps for referring and admitting veterans to a community living center (CLC). Visual figures showing the five consistent processes that occur when a veteran is referred and admitted to a CLC for six community living centers in the VA

Though differences among sites were identified, most had similar processes for admitting veterans to the CLC. Using our process maps (Fig. 1), we were able to identify five consistent processes or behaviors that staff engage in as veterans are referred and admitted to a CLC: assessing, discussing, decision-making, documenting, and re-assessing.

### Assessing

All sites reported that there is an initial assessment that takes place to determine whether a veteran should be admitted to the CLC. This initial assessment was commonly completed by an MD, NP, or PA. One site had an admissions coordinator whose job was to review the veteran’s record before referring to the MD or NP for their review. Another site had an MD and social worker complete the initial review. The CLCs also reported that they completed the MDS 3.0 assessment with all veterans upon admission. National policy requires that the MDS assessment be completed and uploaded to a national VHA database within 14 days of admission.

### Discussing

Sites reported that one of the major goals of the initial assessment process was to discuss the veteran’s preferences and goals for care. At all sites, interdisciplinary teams (IDT) further assessed the patient after admission and discussed next steps of care. The IDT met on average once per week, with one site reporting that they met twice each week. The IDTs were composed of prescribing providers, nurses, mental health staff, nutrition, and rehabilitative staff. They generally discuss all new admissions, and at many CLCs, if possible, they invite the veteran and his/her caregivers to participate in the initial meeting.

### Decision-making

Decision-making occurs at many time points during the admission process to a CLC. The first decision is whether the veteran should be admitted into the CLC. Decisions are made regarding what to write in the assessment and the level of detail that should be reported in the electronic medical record. Another decision that must take place is when to re-assess the veteran. Depending on veteran needs, this could be done regularly during IDT meetings or as part of the larger re-assessment processes mandated by MDS assessments, or at least every 90 days.

Some veterans are admitted to CLCs for short-term rehabilitation or until they regain the ability to care for themselves. These are additional areas that require decision-making on the part of the CLC staff. They must decide if it is most appropriate for a veteran to return home or if they require additional time in the CLC. These discussions and consequent decisions often involve the IDT team and the veteran and/or their caregivers/surrogate decision-makers.

### Documenting

The assessments completed during the admission process must be documented. The MDS is an assessment process that requires mandated documentation to be completed. While the providers are making decisions related to the care of the veteran, they must ensure that these decisions are being documented and are in the correct place. This will enable other providers to view the assessments and decisions that were made.

### Re-assessing

The final consistent process that was identified for veterans who had been admitted into a CLC is the process of re-assessing. The MDS mandates that reassessments take place every 90 days. The IDTs reported that they conduct their own reassessment measure as well and often use the 90-day period mandated by MDS. If a veteran had a change in status prior to the 90-day reassessment, such as a hospitalization, he or she would be re-assessed sooner. One hospice unit within a CLC stated that they complete re-assessment every 2 weeks.

## Discussion

The overarching goal of the LTC QUERI program is to help implement the LSTDI into VHA long-term care settings; by creating and reviewing visual process maps across sites, we learned that adding the GoCC to the admission process would likely be an efficient use of time for both providers and the veteran for several reasons. First, as a veteran is admitted into a CLC following illness, surgery or for longer-term purposes, adding a discussion geared towards assessing the veteran’s current and future care preferences seems most appropriate. Second, veterans in CLCs are often seriously ill, which is the target group for a GoCC. Third, these veterans are already completing a comprehensive admission process, so the addition of a GoCC at this time may be a natural fit. It is important to note that the GoCC could get dismissed because of the number of assessments already taking place during this busy time.

Process mapping enabled us to create a visual representation of the current processes for admitting veterans to the CLC. This representation provided clear information regarding the consistent set of processes and behaviors taking place across all CLCs during admissions, though the timeline and staffing for specific elements may have differed. Our process maps served as the “Current State” in the “You Are Here” process [11]. We depicted the CLC admission process as it was truly operating at the time of our site visit. By viewing the current processes, we were able to identify potential points of entry for a GoCC.

Time constraints on the part of providers are often cited as a possible barrier to completing a GoCC. Incorporating the GoCC into the admissions process increases time efficiency and minimizes the burden on the staff, compared with engaging in these processes after admission is complete. Completing the LST template during the CLC admission process will ensure that the veteran’s care goals are elicited and documented in the electronic medical records in a timely fashion. By completing the GoCC during the current CLC admission process, it has the highest potential for impact on care. The multidisciplinary team is already conducting discipline-specific assessments as part of the admission process, so adding a GoCC may help staff effectively plan for future care for the veteran based on his or her goals.

Our work is a pragmatic example of process mapping techniques, which is lacking in the literature that is more focused on the theoretical use of process maps [12–14]. Our work provided a detailed, real-life example of creating process maps. By showing this information, we attempt to demonstrate that process mapping techniques in implementation research need not be very complex. The creation of process maps is not particularly time consuming and the results of the work can directly inform next steps for implementation by making explicit the behaviors that can be targeted for change through implementation interventions.

We note that process mapping has been used for many years in quality improvement work and is often the initial step when using Lean approaches—mapping the current state as part of the problem analysis [14, 15]. It has also been described as a component of implementation, but using process mapping is still not a common approach within implementation practice and research.

When large systems, such as the VHA, mandate national initiatives, they can be cumbersome and time consuming for facilities to successfully implement. These large initiatives have often been planned and trialed extensively before being released, but it is impossible to account for every individual facility difference that may exist. Using process mapping techniques in care settings for individual facilities allows sites to get a more robust understanding of the current processes taking place and how best to implement the proposed initiative based on their individual institution. It may also facilitate tailoring to the individual facility if needed.

## Limitations

We note that our methods are rapid and not very formal. More formal approaches for developing process maps can yield in-depth insights that are useful, but may not be feasible. Visualizing current processes can help both external groups such as ours, and internal groups, think through places where processes are modifiable and relatively easy to change.

We did not formally record and transcribe our interviews, but we did engage in a process of member checking with the sites after our site visits. We acknowledge that not recording our interviews is a limitation and suggest that research teams planning to utilize process mapping techniques audio record these encounters.

Finally, due to circumstances beyond our control, the LSTDI was significantly delayed in its national rollout. As a result, the initial site visits and interviews we describe in this paper were followed by a relatively long hiatus during which it was not feasible to implement the initiative. Due to the national rollout delay, we found that some providers did not have an extensive understanding of the LSTDI at the time the interviews took place. This limited our ability to deploy the process maps at the time they were developed. The national rollout delay and lack of provider knowledge about the LSTDI impacted our ability to accurately assess provider understanding of the initiative and their perspective on the impact of the initiative on their workflow processes. We consulted with our research team and operational partners on barriers and facilitators to implementation, but our work would have been bolstered if our interviews took place closer to the initial implementation date. If possible, we recommend that research teams conduct interviews to be used for process mapping techniques as close to the implementation date as is feasible in order to address these limitations.

## Conclusions

The use of process maps supports further efforts to implement GoCCs, as they allow us to assess specific processes and behaviors, target individuals whose behavior may need to change to enable the addition of GoCCs to the admission process, and support assessment of barriers and facilitators to implementation success. In future work, we are using these process maps to identify key providers who may be able to support implementation of the LST initiative in CLCs and to design implementation interventions.

## Supplementary Information

**Additional file 1.** Background of the case example.

## Data Availability

Because this work is being done as quality improvement, data will only be available from the authors on request and after approval by the authorizing officials.

## Abbreviations

CLCs: Community living centers
DO: Doctors of osteopathy
GoCC: Goals of care conversations
IDT: Interdisciplinary team
LST: Life-sustaining treatment
LSTDI: Life-Sustaining Treatment Decisions Initiative
LTC QUERI: Long-Term Care Quality Enhancement Research Initiative
MD: Medical doctors
MDS: Minimum data set
NCEHC: National Center for Ethics in Health Care
NP: Nurse practitioners
PA: Physician assistants
QI: Quality improvement
VHA: Veterans Health Administration
VISN: Veterans Integrated Service Networks
