# Correction: Visual process maps to support implementation efforts: a case example

**DOI:** 10.1186/s43058-023-00414-6

**Published:** 2023-03-20

**Authors:** Jennifer Kononowech, Zach Landis-Lewis, Joan Carpenter, Mary Ersek, Robert Hogikyan, Cari Levy, Ciaran Phibbs, Winifred Scott, Anne E. Sales

**Affiliations:** 1 Center for Clinical Management Research, VA Ann Arbor Healthcare System, 2800 Plymouth Rd, Ann Arbor, MI 48109 USA; 2 University of Michigan Medical School, 300 N. Ingalls Street, Ann Arbor, MI 48109 USA; 3 Corporal Michael J. Crescenz VAMC, 3900 Woodland Avenue, Philadelphia, PA 19104 USA; 4 School of Nursing, University of Pennsylvania, 418 Curie Blvd, Philadelphia, PA 19104 USA; 5 VA Ann Arbor Healthcare System, 2215 Fuller Road, Ann Arbor, MI 48105 USA; 6 Eastern Colorado Health Care System, 1055 Clermont St, Denver, CO 80220 USA; 7 School of Medicine, University of Colorado Anschutz Campus, 13001 E. 17th Pl, Aurora, CO 80045 USA; 8 Geriatrics and Extended Care Data and Analysis Center, VA Palo Alto Health Care System, 3801 Miranda Ave, Palo Alto, CA 94304 USA; 9 Department of Pediatrics, Stanford University School of Medicine, 725 Welch Road, Palo Alto, CA 94304 USA

**Correction: Implement Sci Commun 1, 105 (2020)**

**https://doi.org/10.1186/s43058-020-00094-6**

Following the publication of the original article [1] the authors requested to update the “Competing interests” section as follows:

“The authors declare that Anne Sales is co-Editor-in-Chief of the journal."
