# Supplementary material for: Visual process maps to support implementation efforts: a case example
Source: Implement Sci Commun. 2020 Nov 25;1:105. doi: 10.1186/s43058-020-00094-6 (PMC7687814; doi:10.1186/s43058-020-00094-6)
Supplement: Supplementary file 1 — Additional file 1. Background of the case example. [file 43058_2020_94_MOESM1_ESM.docx]

**Background of the case example**

Persons with serious illness receiving care in long-term care settings are often vulnerable, frail and are likely to experience a serious health event in the near future. Long-term care settings provide an opportunity for residents and/or their families to discuss life-sustaining treatments (LST) with their healthcare providers. The purpose of goals of care conversations (GoCC) is to elicit the patients’ values and treatment preferences either from the patient directly or, in the case of patients who lack capacity, from their designated surrogate decision-maker. Patients who have GoCCs with their healthcare providers may experience better quality of life, less aggressive medical care at the end of life, and earlier referral to hospice [1].

The Veterans Health Administration (VHA), the largest integrated healthcare delivery system in the United States, prioritized conducting GoCC and documenting treatment preferences of seriously-ill Veterans. The National Center for Ethics in Health Care (NCEHC) released VHA Handbook 1004.03, “*Life-Sustaining Treatment Decisions: Eliciting, Documenting, and Honoring Patients' Values, Goals and Preferences”* in January 2017 [2]. The Handbook updated guidance for assessing Veterans’ goals, values, and preferences for LST decisions. The aim of this guidance, called the Life-Sustaining Treatment Decisions Initiative (LSTDI) is to promote personalized, proactive, patient-driven care for Veterans with serious illness. The Handbook sets minimum expectations for GoCC to occur when a patient is at high risk of experiencing a life-threatening clinical event within the next 1-2 years. The initiative was expected to be fully implemented in VHA by July 2018.

The NCEHC extensively tested the new LST template with four geographically dispersed sites [3]. These sites volunteered to serve as demonstration sites, provided crucial input on the initiative and helped improve resources to support it. Based on feedback from the demonstration sites, the NCEHC refined the documentation of GoCCs and enhanced the staff education process and patient education resources.

The initiative has an associated LST progress note and order set within the VHA’s electronic medical record system. The progress note must be completed by licensed prescribing providers (providers who are credentialed to write orders, including prescriptions and other orders for care) and documents a GoCC with seriously ill Veterans. A GoCC is used to elicit the Veteran’s values, goals and preferences for care, and based on those factors, the associated template is used to document decisions about initiating, limiting or discontinuing life-sustaining treatments. Previous research has shown that the majority of documented advance care planning discussions, and orders for treatment such as “Do not resuscitate”, are not easily accessible in urgent or emergent situations [4]. To overcome this challenge, the LST progress note and order set were designed to be easily accessible via VHA electronic medical record by all providers. The completed LST progress note is accessible from the cover sheet of the electronic medical record and the associated LST order set defaults to the top of the list on the Orders tab [3].

Many clinicians have a role in conducting and documenting GoCCs. Registered Nurses, Social Workers, Psychologists and Chaplains may have the initial conversation with a Veteran to better understand his/her values and goals. Medical Doctors (MDs), Doctors of Osteopathy (DOs), Physician Assistants (PAs) and Nurse Practitioners (NPs) explain a Veteran’s diagnosis, prognosis, and establish an LST plan with the Veteran and/or their surrogate; as licensed prescribing providers, this group of clinicians are responsible for completing and signing the LST progress note and order set.

Community Living Centers are units within VA that were previously known as nursing home care units [5]. The mission of the CLC is to restore and maintain each Veteran to his or her highest level of well-being. CLCs are heterogeneous units that can provide long-stay nursing home care, short-stay skilled nursing care, and hospice care; not all CLCs provide all of these types of care and there is considerable variation in the types of care that any CLC provides. CLCs attempt to provide a ‘home like’ atmosphere [6-7]. Length of stay in CLCs varies greatly depending on the Veteran’s needs and the individual CLC. Some CLCs provide short-term care following a hospitalization or during treatment, while others provide long-term, residential care. A given VHA facility may have multiple CLC units.

Before Veterans are admitted into a CLC unit they must be assessed to determine if the services provided by the CLC are appropriate. Different approaches to admission decision making are used across VHA, depending on the care needs of the Veteran, bed availability, and local policy. Once admitted, residents are evaluated using the Minimum Data Set (MDS 3.0), a mandatory national tool that was designed to standardize assessment and determine care needs in VA and non VHA nursing homes [8]. The MDS measures health and quality of life including functional status, patient preferences for daily care, mood, symptoms such as pain, and medical conditions [9]. Admissions processes are built around several processes, including the assessments conducted for the MDS.

**Methods in more detail**

Two to four QUERI team members completed site visits to six CLCs in one Midwestern Veterans Integrated Service Network (VISN) between October 2015 and May 2016. VHA divides the United States into 18 VISNs, regional systems of care working together to better meet local health care needs and provide greater access to care. These six sites were chosen because of their proximity to the LTC QUERI staff based in Ann Arbor, MI. The initial goal of the site visit was to develop rapport with a site champion who were identified as CLC leaders who agreed to be liaisons for the project. In addition to the site champion, we also met with and interviewed facility leadership and team members from nursing, quality management, restorative care, and social work. Site visits typically lasted one day each and included a combination of one-on-one meetings and group discussions.

We conducted semi-structured interviews with the site champion and other CLC staff members to understand how Veterans are currently admitted into the CLC and to identify existing or potential process changes that would facilitate conducting GoCC and documenting Veterans’ LST preferences. We queried interviewees to find out which staff members were involved in each part of the admission process. We inquired about the configuration of the CLC, including number of beds and types of units, as these differ based on each VA. We also asked whether sites were already conducting GoCC prior to the national roll-out of the LSTDI. A dedicated note taker was present at every meeting or interview to document the visit process and capture the discussions. Following the meetings, the notes were reviewed by all team members and edited for accuracy. The final version of the site visit notes was reviewed by all project team members, and a summary was sent back to the site champion for review.

One team member (AES) created the initial process maps following our early site visits after analyzing the site visit notes. This map was circulated to the research team for feedback and refinement. For later site visits the project manager (JK) took the lead in drafting the process maps after receiving training from AES in process map methodology. These process maps were created by completing a careful review of the initial process maps alongside the later site visit notes. Similarities and differences among sites were identified and reflected in the process maps. All process maps were circulated to the research team for feedback prior to being finalized. After maps were drafted, they were analyzed for opportunities for process improvement related to implementing GoCCs and LST template completion. We used other information from the interviews to note potential barriers and facilitators to implementation, using the Tailored Implementation for Chronic Diseases checklist [10].

References

1. Bernacki RE, Block SD. Communication about serious illness care goals: A review and synthesis of best practices. JAMA Intern Med. 2014;174:1994–2003.
2. Veterans Health Administration: VHA Handbook 1004.03, Life-Sustaining Treatment Decisions: Eliciting, Documenting, and Honoring Patients’ Values, Goals, and Preferences. www.va.gov/vhapublications/ViewPublication.asp?pub ID = 4308. Accessed 1 February 2017.
3. Foglia MB, Lowery J, Sharpe VA, Tompkins P, Fox E. A comprehensive approach to eliciting, documenting, and honoring patient wishes for care near end of life: The veteran health administration’s life-sustaining treatment decisions initiative. Jt Comm J Qual Patient Saf. 2019;45(1):1-10.
4. Walker E, McMahan R, Barnes D, Katen M, Lamas D, Sudore R. Advance care planning documentation practices and accessibility in the electronic health record: Implications for patient safety. J Pain Symptom Manage. 2018;55(2):256-264.
5. Hojlo C. The VA’s transformation of nursing home care: From nursing homes to community living centers. Generations. 2010;34:43-48.
6. Veterans Health Administration. Rehabilitation Continuum of Care. http://www.va.gov/vhapublications/ViewPublication.asp?pub_ID¼3066.
7. Harrison J, Tyler DA, Shield RR, Mills WL, Morgan KE, Cutty ME, et al. An unintended consequence of culture change in VA community living centers. J Am Med Dir Assoc. 2017;18(4):320-325.
8. Morris JN, Hawes C, Fries BE, Phillips CD, Mor V, Katz S, et al. Designing the national resident assessment instrument for nursing homes. Gerontologist. 1990;30(3):293-307.
9. Saliba D, Buchanan J. Making the investment count: Revision of the minimum data set for nursing. J Am Med Dir Assoc. 2012;13(7):602-610.
10. Ritchie MJ, Dollar KM, Miller CJ, Oliver KA, Smith JL, Lindsay JA, Kirchner JE. Using

implementation facilitation to improve care in the veterans health administration

(Version 2). Veterans Health Administration, Quality Enhancement Research Initiative

(QUERI) for Team-Based Behavioral Health, 2017. Available at:

<https://www.queri.research.va.gov/tools/implementation/Facilitation-Manual.pdf>
